# Cessation of anti-VLA-4 therapy in a focal rat model of multiple sclerosis causes an increase in neuroinflammation

**DOI:** 10.1186/s13550-019-0508-7

**Published:** 2019-05-09

**Authors:** S. K. Vainio, A. M. Dickens, J. Tuisku, O. Eskola, O. Solin, E. Löyttyniemi, D. C. Anthony, J. O. Rinne, L. Airas, M. Haaparanta-Solin

**Affiliations:** 1 Turku PET Centre, Preclinical PET Imaging, University of Turku, Tykistökatu 6 A, 20520 Turku, Finland; 2 MediCity Research Laboratory, University of Turku, Turku, Finland; 3 Turku Centre for Biotechnology, University of Turku, Turku, Finland; 4 Department of Pharmacology, University of Oxford, Oxford, UK; 5 Turku PET Centre, Clinical Neurology, Turku University Hospital, Turku, Finland; 6 Turku PET Centre, Radiopharmaceutical Chemistry Laboratory, University of Turku, Turku, Finland; 7 Department of Chemistry, University of Turku, Turku, Finland; 8 Accelerator Laboratory, Åbo Akademi University, Turku, Finland; 9 Department of Biostatistics, University of Turku, Turku, Finland; 10 Division of Clinical Neurosciences, Turku University Hospital, Turku, Finland; 11 Department of Clinical Medicine, University of Turku, Turku, Finland

**Keywords:** Multiple sclerosis, Anti-VLA-4, EAE, Positron emission tomography, TSPO, [^18^F]GE-180

## Abstract

**Background:**

Positron emission tomography (PET) can be used for in vivo evaluation of the pathology associated with multiple sclerosis. We investigated the use of longitudinal PET imaging and the 18-kDa translocator protein (TSPO) binding radioligand [^18^F]GE-180 to detect changes in a chronic multiple sclerosis-like focal delayed-type hypersensitivity experimental autoimmune encephalomyelitis (*f*DTH-EAE) rat model during and after anti-VLA-4 monoclonal antibody (mAb) treatment. Thirty days after lesion activation, *f*DTH-EAE rats were treated with the anti-VLA-4 mAb (*n* = 4) or a control mAb (*n* = 4; 5 mg/kg, every third day, subcutaneously) for 31 days. Animals were imaged with [^18^F]GE-180 on days 30, 44, 65, 86 and 142. Another group of animals (*n* = 4) was used for visualisation the microglia with Iba-1 at day 44 after a 2-week treatment period.

**Results:**

After a 2-week treatment period on day 44, there was a declining trend (*p* = 0.067) in [^18^F]GE-180-binding in the anti-VLA-4 mAb-treated animals versus controls. However, cessation of treatment for 4 days after a 31-day treatment period increased [^18^F]GE-180 binding in animals treated with anti-VLA-4 mAb compared to the control group (*p* = 0.0003). There was no difference between the groups in TSPO binding by day 142.

**Conclusions:**

These results demonstrated that cessation of anti-VLA-4 mAb treatment for 4 days caused a transient rebound increase in neuroinflammation. This highlights the usefulness of serial TSPO imaging in the *f*DTH-EAE model to better understand the rebound phenomenon.

## Background

Multiple sclerosis (MS) is an inflammatory disease characterised by neurodegeneration and demyelination [1, 2]. Its diagnosis is based primarily on clinical symptoms, focal abnormalities in magnetic resonance imaging (MRI), and evidence of inflammatory markers in the cerebrospinal fluid [3]. Acute inflammation subsides with advancing disease, and diffuse pathology that is as yet undetectable by MRI often prevails. This is associated with gradual, progressive increases in clinical disability status [4]. These observations highlight the need for the development of new tools for in vivo evaluation of diffuse MS pathology that cannot be detected by MRI. Positron emission tomography (PET) allows the observation of diffuse inflammatory changes in MS. This is achieved using microdoses of radioligands that can cross the blood-brain barrier (BBB) and selectively target inflammatory markers that are not accessible to high-concentration MRI-visible, intravascular contrast agents [5, 6]. PET imaging of the diffuse microglial activation associated with progressive MS pathology can be achieved using radioligands that bind to the 18-kDa translocator protein (TSPO), which is upregulated in activated microglial cells and astrocytes [7].

Experimental autoimmune encephalomyelitis (EAE) is widely used as a model of MS [8], but the small focal lesions that are disseminated along the neuraxis have proven difficult to image and quantify by PET imaging. The use of focal delayed-type hypersensitivity EAE (*f*DTH-EAE), which is induced by stereotactic injection of the antigen to a given location within one hemisphere of the animal brain [9], elicits larger, predictable lesions that can be visualised in the rodent brain by TSPO-based PET imaging. This focal model conserves many of the most prominent hallmarks of MS pathogenesis including the recruitment of monocytes and T cells, axonal and myelin damage and, importantly, microglial activation [9–11]. In addition, the presence of a single lesion at a specific location allows longitudinal assessment of lesion activity even after the BBB has resealed [12]. Typically, the *f*DTH-EAE lesions are in brain structures, such as in the striatum, to ensure that there are no overt clinical signs, which are often severe in the disseminated models and which give rise to comorbidities and then to death [13].

The monoclonal antibody (mAb) natalizumab binds to the very late antigen-4 (VLA-4) integrin [14], and natalizumab treatment of relapsing-remitting MS (RRMS) is a more effective therapy than most other available therapies [15]. Natalizumab was initially developed when studies of disseminated EAE demonstrated that anti-VLA-4 mAbs were efficacious in slowing disease development by preventing the entry of encephalitogenic T cells into the central nervous system (CNS) [16]. Despite the clinical success of anti-VLA-4 mAbs, treatment must often be withdrawn. In clinical studies, the removal of anti-VLA-4 treatment returns the patient to a pre-natalizumab state, and there is a rebound risk of severe relapse [17–20]. Thus, it is important to understand how the cessation of VLA-4-vascular cell adhesion molecule 1 (VCAM-1) blockade impacts the diffuse microglial activation that is observed in the progressive phase of *f*DTH-EAE and whether cessation results in overall worsening of the pathology in the longer term. Studies using conventional models of MS have proven inconclusive. Treatment of EAE with anti-VLA-4 mAb at different phases of disease has discordant effects: while it exacerbates ongoing relapsing EAE, early treatment of progressive EAE prevents inflammatory infiltrates [21, 22].

Here, to overcome the limitations of current EAE models, we sought to investigate the effect of a start-stop regime of anti-VLA-4 treatment on the chronic *f*DTH-EAE model using longitudinal PET imaging with an improved TSPO radioligand [^18^F]GE-180 when compared to [^11^C]PK11195 [23].

## Materials and methods

### Animals

Animal experiments received ethics approval from the Finnish National Animal Experiment Board (ESAVI/6360/04.10.03/2011). Male Lewis rats (*n* = 12) were obtained from Charles River Laboratories, Germany and allowed to acclimatise 7 days before the start of the experiments. All animals were housed in accordance with the Amsterdam protocol for animal experiments in individually ventilated cages with a mean consistent temperature of 21 (1.2) °C and consistent humidity of 55 (5)% with a 12-h light/dark cycle. Food (CRM(E) Expanded, Special Diet Services, UK) and tap water were provided ad libitum.

### Animal model

*f*DTH-EAE was induced by stereotaxic injection of Bacille Calmette-Guérin (BCG) into the striatum, and by subsequent peripheral activation of the lesion using complete Freund’s adjuvant, as described previously [11].

Briefly, on day − 28 and before the procedure, an opioid drug (Temgesic 0.3 mg/mL, Reckitt-Benckiser Healthcare, UK; 0.05 mg/kg, intraperitoneal) was given to the rats for analgesia, then they were anaesthetised with isoflurane (Virbac Animal Health, Carros, France) mixed with air (induction 4%, 700 mL/min and maintenance 2.5%, 400 mL/min) and placed onto a stereotaxic frame (Kopf Instruments, USA). The stereotaxic frame was positioned on an electric heating pad to keep the body temperature of the animal stable during the operation. Oftagel (2.5 mg/g, Santen, Tampere, Finland) was applied to their eyes to keep them from drying during anaesthesia. A burr hole (1.5 mm) was drilled in the skull with a dentist’s drill in a position that was determined relative to the bregma (antero-posterior + 1 mm; lateral − 3 mm; dorsal-ventral − 4.0 mm) [24]. Heat-killed BCG (1 × 10^5^ cells in 2 μL of PBS) was injected into the left caudate putamen with a Hamilton syringe (10 μL). Next, 28 days following the intracerebral injection of BCG on day 0, the lesion was activated by the intradermal injection of M. tuberculosis (1.5 mg, Difco Laboratories, Detroit, MI, USA) in complete Freund’s adjuvant (100 μL, Sigma Aldrich, Saint Louis, MO, USA) in the animal’s flanks.

**Fig. 1:**
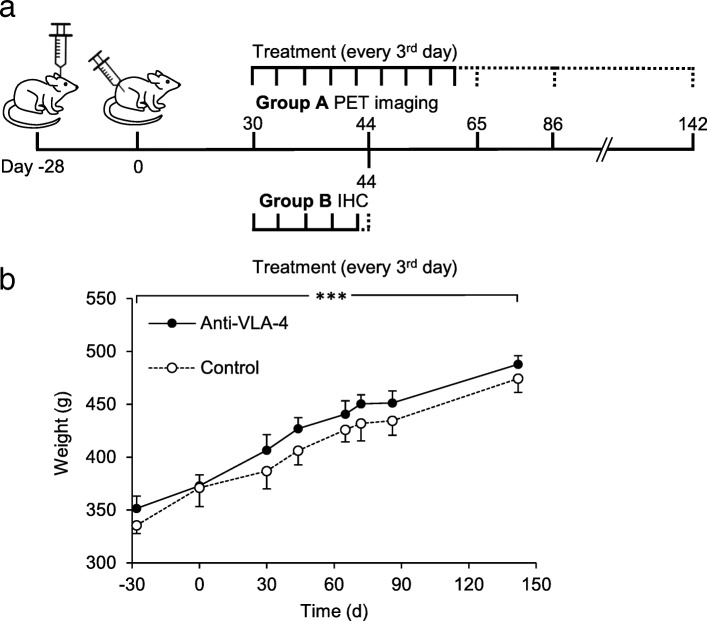
A schema of the experimental design showing the timeline of the procedures and changes in the rats’ body weight during the study. **a** Stereotaxic operation with BCG was performed on day − 28 (group A, *n* = 8 and group B, *n* = 4). Activation of the lesion was performed on day 0. Animals in group A were treated every third day with anti-VLA-4 mAb (*n* = 4) or with an isotype matched control mAb (*n* = 4) on days 30–61. The dashed line indicates the follow-up period after treatment. PET images were acquired on days 30, 44, 65, 86 and 142. Animals for IHC were treated every third day during days 30–44 and were killed on day 44 (group B). **b** Changes in the body weight of the animals during the PET imaging study. Results are shown as means (SD). ***p < 0.001; *BCG* Bacille Calmette-Guérin, *IHC* immunohistochemical, *mAb* monoclonal antibody, *PET* positron emission tomography

The animals were divided into two different groups: group A was used to evaluate anti-VLA-4 mAb treatment using TSPO PET imaging until day 142 (*n* = 8), and group B was used for immunohistochemical (IHC) evaluation of the chronic lesion on day 44, after 14 days of treatment with anti-VLA-4 mAb (*n* = 4: anti-VLA-4 treated *n* = 2, control *n* = 2) (Fig. 1a).

### Anti-VLA-4 treatment

The animals (*n* = 12) were injected intrastriatally with the antigen on day − 28, and peripheral activation was performed on day 0 (Fig. 1a). Treatment with anti-rat VLA-4 mAb (GG5/3 Ab, ELAN Pharmaceuticals, USA) or the matched isotype control mAb (JH70 6F10, ELAN Pharmaceuticals, USA) was started 30 days after the peripheral activation of the lesion to represent a chronic state of the lesion. Anti-VLA-4 (5 mg/kg) or control mAb (5 mg/kg) [21, 22] were administered by subcutaneous (sc) injection every third day during the 30–61 day period (group A) or during the 30–44 day period (group B) (Fig. 1a).

### Radiotracer production

[^18^F]GE-180 was synthesised at the Radiopharmaceutical Chemistry Laboratory of Turku PET Centre as described previously [25]. The molar activity of [^18^F]GE-180 was 2.0 (0.5) TBq/μmol at the end of synthesis (*n* = 5). Radiochemical purity was ≥ 98%.

### In vivo PET/CT imaging

Animals from group A (*n* = 8: four anti-VLA-4 mAb-treated rats and four isotype-matched control mAb-treated rats) were imaged on days 30, 44, 65, 86 and 142 after peripheral activation of the lesion (Fig. 1a). Body weight was measured right before each PET scan (Fig. 1b).

Dynamic in vivo imaging was performed with a Siemens Inveon Multimodality PET/CT (Siemens Medical Solutions, USA) small animal scanner that generates images with 159 transaxial slices with a 10 cm transaxial and a 12.7 cm axial field of view. Prior to imaging, each animal was anesthetised with isoflurane mixed with oxygen (induction 4%, 700 mL/min; maintenance 2.5%, 400 mL/min). Two rats were imaged simultaneously during each imaging session. Body temperature was maintained using an electric heating pad, and Oftagel (2.5 mg/g, Santen) was applied to the eyes. The scan was initiated immediately after an intravenous (iv) injection of [^18^F]GE-180. The average injected dose was 43.1 (6.7) MBq for treated animals and 42.2 (6.1) MBq for control animals. For the anti-VLA-4-treated animals, the injected mass was 36.8 (5.0) ng/kg (0.106 (0.014) nmol/kg) and 42.0 (15.7) ng/kg (0.121 (0.045) nmol/kg) for the control animals.

A computed tomography (CT) scan was performed in order to correct for signal attenuation in the PET scan and to provide anatomical context. Emission scans (45 min) were acquired in list mode with an emission window of 350 to 650 MeV with framing 30 × 10 s, 15 × 60 s, 5 × 300 s.

The data were reconstructed using the ordered-subsets expectation maximisation algorithm in three dimensions (OSEM3D) two times and MAP iterative reconstruction protocols 18 times in the InveonTM acquisition software (Siemens Medical Solutions, USA).

### PET analyses

Simplified reference tissue modelling has been used previously to analyse TSPO tracers with contralateral reference input [26]. In the *f*DTH-EAE-model, the input can be obtained from the contralateral hemisphere due to the low density of the microglia [9], and thus, due to low non-specific radioligand binding. Reference regions have been evaluated in clinical settings, and no anatomically distinct reference region that is free of pathology was found in the brains of MS patients [6]. Here, PET and CT image pre-processing was carried out with PMOD analysis software (v3.4, PMOD Technologies Ltd., Zurich, Switzerland) by first dividing image files into single-animal images and then cropping the image to the skull area. The CT image was first aligned with the summed PET image using the rigid matching tool and was subsequently aligned with the inbuilt Schiffer rat MRI template image. These two transformations were combined to align the dynamic PET image with the MRI template image. Next, the non-displaceable binding potential (BP_ND_) maps were estimated with an in-house created basis function implementation of a simplified reference tissue model [27] in MatLab 2011 (The MathWorks, Natick, MA, USA). A uniform spherical volume of interest, with the size of 0.014 cm^3^ on the contralateral hemisphere, was used as a reference tissue input for the model. The parameter θ was limited to intervals from 0.05 to 0.4 min^− 1^, and 100 basis functions were used in the estimation of the BP_ND_. The regional mean BP_ND_ was determined from the BP_ND_ map using the iso-contour tool, where the threshold BP_ND_ value for the lesion area was manually chosen by visual inspection. The change in BP_ND_ was calculated by normalising the results to the day 30 results by calculating the ratio between the value on day X (i.e., day 44, 65, 86, or 142) and the value on day 30.

### Immunohistochemistry and histology

Animals in group B (*n* = 4) were used for IHC analysis on day 44 after 14 days of treatment. Animals were euthanised with an overdose of sodium pentobarbital (Mebunat vet 10 mg/100 g, intraperitoneal injection, Orion Pharma, Turku, Finland). The blood was then removed via cardiac puncture and perfused first with heparinised saline with paraformaldehyde-lysine-periodate light fixative containing 0.1% glutaraldehyde. The brains were cryoprotected in 30% (*w*/*v*) sucrose solution. IHC staining was performed using an antibody (Ab) against the ionised calcium-binding adapter molecule 1 (Iba-1, Wako 019-19741, Rabbit-anti-rat, Wako Chemicals GmbH, Neuss, Germany) to detect microglia. The sections were incubated with anti-Iba-1 primary Ab overnight at 4 °C. After this, the sections were incubated with anti-rabbit IgG (Invitrogen, Camarillo, CA, USA) for 1 h at RT. Finally, they were incubated with avidin-biotin-peroxidase complex (Vectastain Elite kits, Vector Laboratories, Burlingame, CA, USA). The sections were stained with 3,3′-diaminobenzidine (SIGMAFAST, Sigma-Aldrich, Saint Louis, MO, USA) and counterstained with cresyl violet to stain the nuclei.

### Statistical analysis

All statistical tests were performed as two-sided tests with the statistical significance level set to 0.05. Statistical analysis was performed using a linear mixed model with compound symmetry covariance structure, including one within-factor (time; indicating overall mean change between baseline and other measurements), one between-factor (group; anti-VLA-4 and control) and an interaction term (group*time). Values for animal weight data, injected radiochemical masses and BP_ND_ are reported as means (SDs).

The interaction examined whether the mean change during the study was different between the treated and control animals in group A. While the interaction was statistically significant, contrasts were calculated between every two time points to determine where the mean changes differ from each other. In addition, the model gave mean estimates for each time point, and comparisons between the groups could be made at each time point. The normality assumption was checked from studentised residuals. The analyses were performed using SAS System, version 9.4 for Windows (SAS Institute Inc., Cary, NC, USA).

## Results

### Anti-VLA-4 treatment did not affect body weight

During the course of the experiment, we observed no adverse effects, such as sickness or motor abnormalities. Both treated and control animals showed similar increases in body weight throughout the experimental period (anti-VLA-4 39%, Ctrl 41%, *p* < 0.0001), and weight gain did not differ between the anti-VLA-4-treated and the control animals (Fig. 1b).

### Cessation of anti-VLA-4 treatment causes an increase in neuroinflammation

**Fig. 2:**
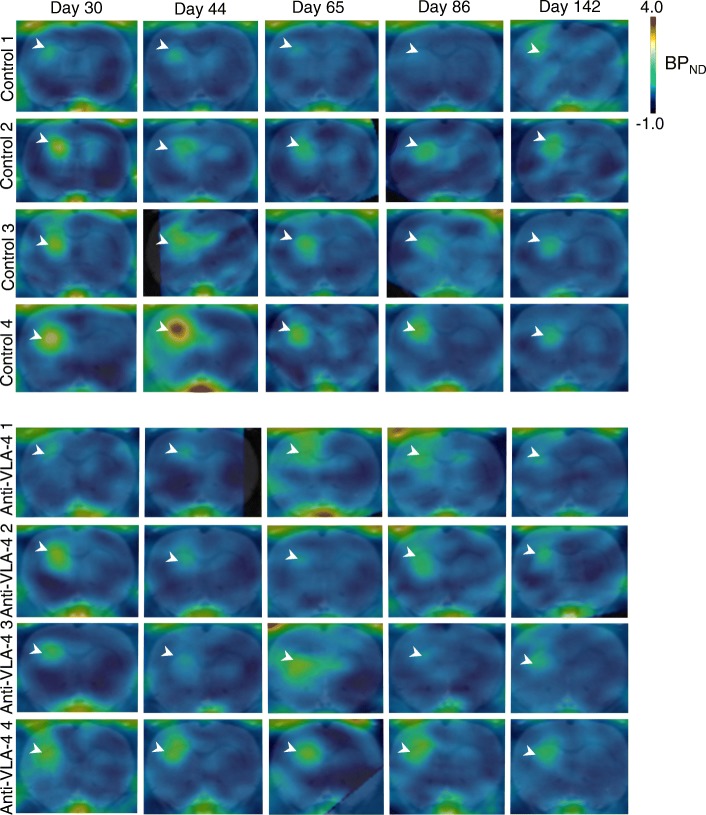
Time course of *f*DTH-EAE in PET imaging of rat brains. Representative coronal images of the BP_ND_ maps modelled with simplified reference tissue modelling and overlaid on a Schiffer MRI template. The control animals (*n* = 4) are shown in the top panels. The anti-VLA-4 mAb treated animals (*n* = 4) are shown on day 30 (when treatment was started), day 44, day 65 (at which time the treatment had been stopped for 4 days), day 86 and day 142. White arrow-heads point at the lesion. *BCG* Bacille Calmette-Guérin, *BP*_*ND*_ non-displaceable binding potential, *fDTH* focal delayed-type hypersensitivity, *EAE* experimental autoimmune encephalomyelitis, *mAb* monoclonal antibody, *MRI* magnetic resonance imaging, *PET* positron emission tomography

**Fig. 3:**
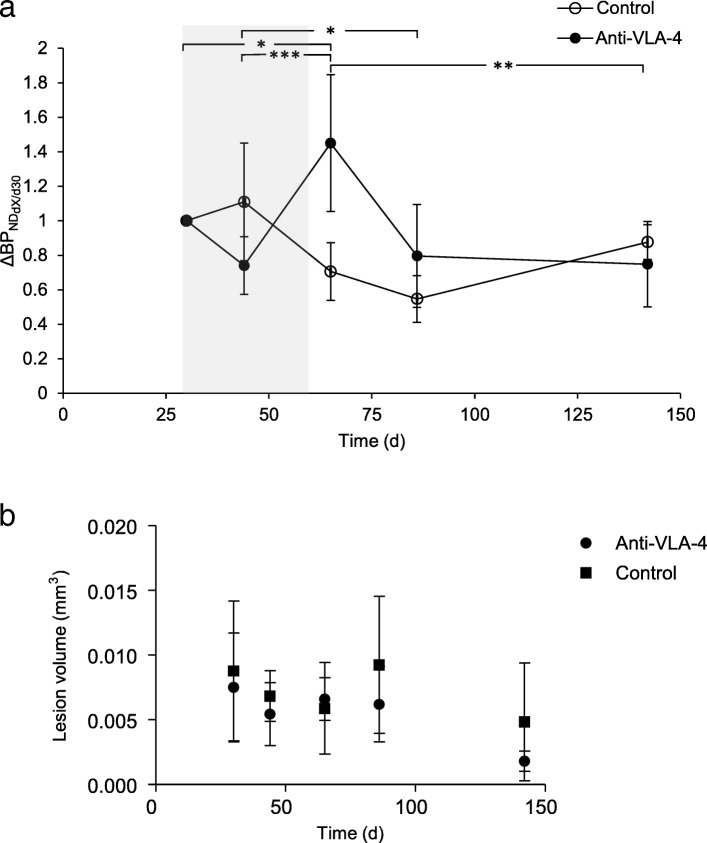
Cessation of anti-VLA-4 mAb treatment leads to rapid activation of microglia, which can be quantified using in vivo TSPO imaging. **a** A graph showing BP_ND_ calculated as a function of treatment by subtracting the day 30 BP_ND_ value from the values at the other time points. Treatment was started 30 days after the activation of the lesion, and a baseline PET/CT image was acquired. Animals were treated with subcutaneous injection of an anti-VLA-4 mAb or an isotype-matched non-binding control mAb every third day until day 61. The treatment period is shown as a light grey background. PET/CT images were acquired on days 30, 44, 65, 86 and 142. Statistical analysis indicates the difference in the mean change during the study between the treated and control animals in group A. All data are plotted as means, with the error bars showing SD values. **b** No changes in lesion volume were detected between treated and control animals. *p < 0.05, **p < 0.01, ***p < 0.001; *BP*_*ND*_ non-displaceable binding potential, *CNS* central nervous system, *CT* computer tomography, *mAb* monoclonal antibody, *MRI* magnetic resonance imaging, *mAb* monoclonal antibody, *PET* positron emission tomography, *VLA*-*4* very late antigen-4

Previous MRI studies have demonstrated that *f*DTH-EAE causes a breakdown in the BBB on day 14. This breakdown resolves by day 28, and the lesion becomes invisible on MRI [10], suggesting that the lesion has developed into a more progressive state [11]. We thus decided to start the anti-VLA-4 treatment on day 30 after lesion activation (after the BBB had resealed) when neuroinflammatory processes continued within the lesion. Following the intrastriatal injection of BCG and the subsequent peripheral activation of the lesion in group A animals, *f*DTH-EAE lesions were detected 30 days after activation of the lesion in baseline PET images (Fig. 2). Two weeks of mAb treatment between days 30 and 44 had effect on [^18^F]GE-180 binding in the anti-VLA-4-treated animals compared to the controls, although on day 44, there was a trend towards decreased binding in the treated animals (*p* = 0.067, Fig. 3a). However, when we compared binding on day 44 to that on day 65, we found a rebound increase in [^18^F]GE-180 binding in animals treated with anti-VLA-4 mAb compared to control animals (*p* = 0.0003). There was also an increase in [^18^F]GE-180 binding between days 30 and 65 (*p* = 0.014). Notably, treatment ended 4 days before day 65. In addition, there was a difference between the treated and control animals in the binding on days 44–86 (*p* = 0.018), with [^18^F]GE-180 binding decreasing over time in the control animals. After the peak in [^18^F]GE-180 binding on day 65 in the animals treated with anti-VLA-4 mAb, the [^18^F]GE-180 binding returned to the level seen in control animals by day 142 (*p* = 0.007) (Fig. 3a). No changes in lesion volume were detected between the control and treated animals (Fig. 3b).

### Anti-Iba-1 showed microglial activation at the lesion site

**Fig. 4:**
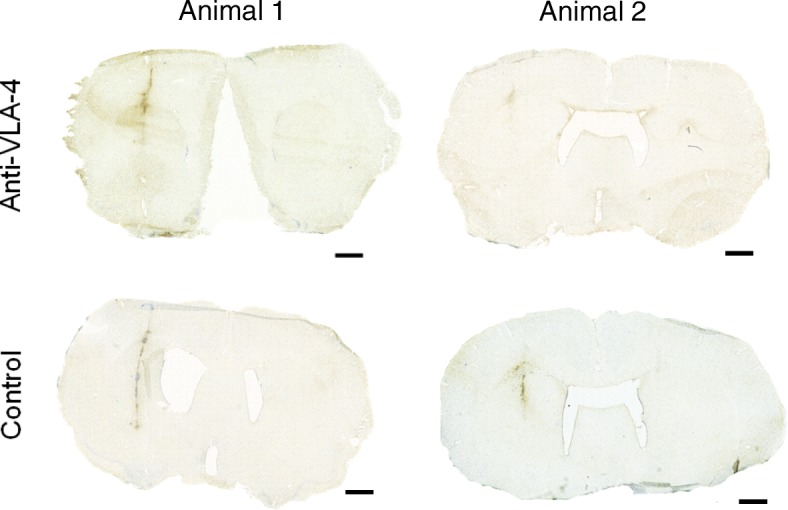
IHC staining with anti-Iba-1 of anti-VLA-4 mAb-treated and control animals after treatment over 14 days. Stereotaxic injection of BCG was performed on day − 28 (*n* = 4), and activation of the lesion was performed on day 0. Anti-VLA-4-treated animals were treated every third day on days 30–44 with anti-VLA-4 mAb (*n* = 2), and the control animals were treated with an isotype-matched control mAb (*n* = 2). The scale bar in each figure is 1500 μm. *BCG* Bacille Calmette-Guérin, *Iba*-*1* ionised calcium-binding adapter molecule 1, *IHC* immunohistochemical, *mAb* monoclonal antibody, *VLA*-*4* very late antigen-4

To confirm the lesions seen on the PET imaging, IHC analysis of Iba-1 expression indicated that there was an infiltrating lesion at the injection site after the stereotaxic injection of BCG and the subsequent peripheral activation of the lesion (Fig. 4).

## Discussion

This study investigated the effect of anti-VLA-4 mAb treatment in a chronic focal DTH-EAE-model and asked whether the TSPO-PET radiotracer [^18^F]GE-180 could be used to non-invasively monitor neuroinflammatory changes during treatment. The results showed that cessation of anti-VLA-4 treatment for 4 days increased [^18^F]GE-180 binding, indicating that there was an increase in neuroinflammation. However, on day 44 after a 2-week treatment period, anti-VLA-4 mAb treatment had only a modest effect on neuroinflammation in the brain. This study involved a small number of animals, but we compensated for this by following the animals longitudinally with PET imaging.

Anti-VLA-4 therapy has shown differential effects in EAE. One study demonstrated that treatment with anti-VLA-4 mAb before clinical disease onset inhibited disease onset, but it did not interfere with peripheral T cell activation [21]. Further, treatment of ongoing relapsing EAE exacerbated the relapses. Mindur and co-workers [22] discovered that early therapy with anti-VLA-4 mAb significantly reduced the severity of progressive EAE, while treatment initiated at an advanced stage was less efficacious. In our study, treatment of a chronic *f*DTH-EAE lesion for 14 days showed a trend towards reduced binding of [^18^F]GE-180, but significant reduction was not detected. This effect might depend on several factors, including the inter-subject variability within the *f*DTH-EAE animals observed in our study. However, the non-significant result in the chronic *f*DTH-EAE lesion after a 2-week treatment is in line with the ASCEND clinical study. This clinical study found that anti-VLA-4 mAb treatment did not delay ambulatory disability or delay disease progression when measured with the expanded disability status scale score, but that it slowed the progression of upper extremity disability in secondary progressive MS. This indicates that the therapy is not effective at a chronic stage of the disease in all measured endpoints, and authors state that treatment effect might be independent of active brain lesions  [28, 29].

There are many reasons for discontinuing anti-VLA-4 mAb therapy in patients, such as the increased risk of progressive multifocal leukoencephalopathy [30] or pregnancy planning [31]. Several clinical case studies report disease activity rebound after anti-VLA-4 mAb therapy cessation [18, 32]. However, the results of cohort studies have been controversial. A study of 375 patients did not confirm the rebound effect, which was defined as relapse activity that was greater than that observed prior to treatment [19]. In contrast, in a cohort of 715 patients, cessation of treatment raised the risk of a severe relapse [20]. Furthermore, 10% of 110 patients showed rebound activity during a high risk period (2 to 8 months after discontinuation of treatment) despite alternative treatments [17]. Cognitive impairment reoccurred along with the radiological and clinical symptoms [33].

To date, increased inflammation after anti-VLA-4 mAb therapy cessation has not been reported in animal models. The *f*DTH-EAE model can be used for longitudinal detection of alterations in inflammation, because the exact location of the lesion is known. In addition to the increase in binding of [^18^F]GE-180 on day 65, a reduction of [^18^F]GE-180 binding to the level of the controls was detected by day 142, which suggests that the increase of [^18^F]GE-180 binding is a transient effect after treatment cessation.

This study used the *f*DTH-EAE model, in which active inflammation peaks after 2 weeks of lesion activation. This is reflected by gadolinium enhancement and BBB breakdown, followed by BBB resealing, typically 4 weeks after lesion activation [10, 34]. Without lesion activation, BCG would remain sequestered behind the closed BBB [9]. The lesion changes and enlarges over time, evolving into a chronic active lesion with abundant microglial activation and axonal damage [11]. In our study, *f*DTH lesions were allowed to evolve until day 30 to mimic a chronic disease state. Previous studies describe hyperintense regions on T2-weighted MRI as well as gadolinium enhancement 2 weeks after lesion activation, indicating BBB breakdown [10, 34]. By day 31, T2 hyperintensity decreases and the area showing gadolinium enhancement is reduced [10], suggesting complete restoration of the BBB. However, tissue damage, including neurofilament loss, occurs behind the sealed BBB [34]. These findings are in accordance with observations in human MS lesions [35]. One hypothesis to explain the increased [^18^F]GE-180 binding on day 30 is that repair mechanisms are activated after damage to the CNS [36]. Furthermore, astrocyte reactivity might result in astrogliosis [37]. A sham-operated group injected with saline was left out from our study, because it has been previously shown that no increased radiotracer binding can be seen after intrastriatal injection of saline [23]. Interpretation of the TSPO imaging data has a limitation in that one cannot distinguish between events that are damaging versus reparative events [38, 39]. TSPO expression reflects the total sum of all neuroinflammatory processes, regardless of whether the processes are involved in damage or repair. TSPO radiotracers are not specific to microglia, and they also bind to reactive astrocytes [23, 40]. Additionally, TSPO binding values can remain significantly above baseline values for up to 3 weeks after CNS damage [41], which might indicate a delay in TSPO expression.

## Conclusion

In conclusion, TSPO PET imaging using [^18^F]GE-180 can detect an increase in neuroinflammation after cessation of anti-VLA-4 mAb treatment in a focal rat model of DTH-EAE. This increase in neuroinflammation was only temporary, as the TSPO signal returned to the levels observed in the control animals by day 142. In addition, an increase in TSPO binding was observed 30 days after lesion induction after the BBB had resealed.

## Abbreviations

[^18^F]GE-180: [^18^F]-*N*,*N*-diethyl-9-(2-fluoroethyl)-5-methoxy-2,3,4,9-tetrahydro-1-H-carbazole-4-carboxamide
Ab: Antibody
BBB: Blood-brain barrier
BCG: Bacille Calmette-Guérin
BP_ND_: Non-displaceable binding potential
CNS: Central nervous system
CT: Computed tomography
DTH: Delayed-type hypersensitivity
EAE: Experimental autoimmune encephalomyelitis
*f*DTH-EAE: Focal delayed-type hypersensitivity experimental autoimmune encephalomyelitis
Iba-1: Ionised calcium-binding adapter molecule 1
IHC: Immunohistochemical
mAb: Monoclonal antibody
MRI: Magnetic resonance imaging
MS: Multiple sclerosis
PET: Positron emission tomography
TSPO: 18-kDa translocator protein
VCAM-1: Vascular cell adhesion molecule 1
VLA-4: Very late antigen-4
